# Construction and evaluation of a *Salmonella* minicell-based dendritic cell-targeted multi-epitope vaccine against *Helicobacter pylori*

**DOI:** 10.3389/fimmu.2025.1595096

**Published:** 2025-06-05

**Authors:** Jiaxue Yang, Kehan Chen, Yalan Zhu, Tiancheng Xie, Chubin Fang, Chuan Wang, Tian Tang

**Affiliations:** ^1^ Department of Laboratory Sciences, West China School of Public Health and West China Fourth Hospital, Sichuan University, Chengdu, Sichuan, China; ^2^ Translational Preventive Medical Research Institute, West China School of Public Health and West China Fourth Hospital, Sichuan University, Chengdu, Sichuan, China

**Keywords:** *Helicobacter pylori*, *Salmonella* minicells, vaccine design, immune efficacy, multi-epitope, stomach cancer

## Abstract

**Background:**

*Helicobacter pylori (H. pylori)* infection is a global health concern linked to various gastrointestinal diseases, highlighting the urgent need for effective vaccines.

**Methods:**

In this study, we developed two multi-epitope vaccine candidates based on *Salmonella* minicells: TA-2m and Apt-TA-2m. Apt-TA-2 is an advanced formulation of TA-2m, coated with dendritic cell-targeting RNA aptamer to enhance antigen delivery and immune activation. The physical properties of the vaccines, including shape, size, particle dispersion index (PDI), and zeta potential, were characterized using transmission electron microscopy (TEM) or dynamic light scattering (DLS). Comprehensive *in vitro* and *in vivo* evaluations were conducted to assess their safety, immunogenicity, and protective efficacy.

**Results:**

Both vaccines demonstrated excellent safety profiles and elicited strong immune responses, significantly reducing *H. pylori* colonization and alleviating gastric pathology. Notably, Apt-TA-2m demonstrated superior immunogenicity, characterized by enhanced T-cell cytokine production, increased mucosal IgA levels, and greater reductions in gastric bacterial loads.

**Conclusions:**

These findings underscore the potential of minicell-based vaccines for combating *H. pylori* infections. The enhanced protective efficacy of the Apt-TA-2m vaccine positions it as a promising candidate for further clinical development.

## Introduction

*Helicobacter pylori* (*H. pylori*) is a Gram-negative bacterium that colonizes the gastric mucosal epithelium, adhering to epithelial cells and releasing toxins that induce chronic inflammation (1, 2). It is highly prevalent, the global infection rate in adults is about 43.9% (3). In China, infection rates among adults vary widely, ranging from 34.2% to 72.3% (4, 5). *H. pylori* is associated with various gastrointestinal disorders, including gastritis, gastric and duodenal ulcers, and mucosal atrophy (1, 6). Additionally, it is a major risk factor for gastric cancer and mucosa-associated lymphoid tissue (MALT) lymphoma (6–8). As a result, the World Health Organization (WHO) classified *H. pylori* as a Group I carcinogen in 1994 (9, 10).

Under natural infection, the human immune system struggles to eliminate *H. pylori* because the bacteria employ toxins to suppress T-cell and dendritic cell activity, while regulatory T cells secrete cytokines to dampen immune responses in children (11, 12). Phase variation of lipopolysaccharide (LPS) further enables *H. pylori* to evade immune recognition (12, 13). Additionally, bacterial defenses, such as urease production, inhibit neutrophil phagocytosis, and weak Th1/Th17 responses fail to clear the infection (12). Consequently, current treatments rely heavily on pharmacological interventions, such as proton pump inhibitors combined with antibiotics (14–16). However, increasing antibiotic resistance has significantly reduced the effectiveness of these treatments, leading to higher reinfection rates (17, 18). To address these challenges, developing a vaccine for both *H. pylori*-negative individuals and those who have cleared the infection through drug therapy is essential. A vaccine offers a promising long-term solution by stimulating robust immune responses, e.g., Th1 and Th17 responses, to reduce *H. pylori* colonization and prevent reinfection, thereby lowering infection rates and improving public health outcomes.

Several strategies have been pursued to develop vaccines against *H. pylori*, including whole cell preparations (19–21), live vectored platforms (22–24), and protein-based formulations (25, 26). Whole cell and live vaccines, though inactivated or attenuated, still contain pathogen derived elements that may elicit undesirable reactions in the host. Protein-based vaccines tend to offer a safer profile but often struggle with inconsistent immunogenicity and occasional side effects stemming from the complex nature of protein antigens (18). In response, multi-epitope vaccines have gained attention for combining safety with enhanced efficacy (27). By strategically selecting and incorporating specific T-cell and B-cell epitopes from immunogenic antigens, multi-epitope vaccines induce a more targeted immune response, minimizing off-target effects and optimizing the activation of relevant immune pathways (28, 29). Furthermore, the reduced size and complexity of multi-epitope constructs facilitate more efficient production and improve stability (30). Despite these advantages, the success of such vaccines heavily depends on the delivery platform used.

Bacterial minicells, small non-dividing nanoparticles (100~500 nm) originating from most bacterial species due to premature septation, offer unique advantages as vaccine delivery carriers (31). They can be engineered to package plasmid DNA or recombinant proteins, making them highly versatile for drug and protein delivery (32–34). Additionally, minicells retain the structural components of their parental bacterial cells, including lipopolysaccharide (LPS), which can be recognized by antigen-presenting cells (APCs) via pathogen-associated molecular patterns (PAMPs) (33). This interaction is essential for generating a robust immune response (35). Due to these advantages, minicell has been increasingly used as a carrier for vaccine development. For instance, Giacalone et al. utilized minicells derived from *E coli* to develop a vaccine capable of simultaneous delivery of plasmid DNA and protein antigens. The minicells were engineered to encapsulate both green fluorescent protein (GFP) and eukaryotic expression plasmids, demonstrating effective induction of antigen-specific immune responses in mice (36). Similarly, Giacalone et al. employed *E. coli*-derived minicells to deliver plasmid DNA encoding the protective antigen of *Bacillus anthracis* to mammalian cells, resulting in robust antibody responses and partial protection against anthrax challenge in mice, highlighting the potential of minicells for genetic vaccine delivery (37). Furthermore, Carleton et al. engineered *Salmonella typhimurium* (*S. typhimurium*) minicells with a type III secretion system to deliver a chimera harboring the *Listeria monocytogenes* MHC class I-restricted immunogenic peptides, eliciting strong CD8+ T-cell responses in BALB/c mice, demonstrating the efficacy of minicells in stimulating cellular immunity (31). These studies collectively underscore the versatility and immunogenicity of minicell-based vaccines.

In this study, the MHC class II-restricted T-cell epitopes, B-cell epitopes, and peptide fragments enriched with both types of epitopes derived from the major antigenic proteins of *H. pylori*, including adhesin A (HpaA), vacuolating cytotoxin A (VacA), and urease subunit beta (UreB), were selected and used to construct a chimeric antigen for vaccine development (38–40). The delivery vehicle for this vaccine was minicells derived from a *S. typhimurium minD* mutant strain TA-1. To generate the minicell-based vaccine against *H. pylori*, the encoding gene of the chimeric antigen fused with the secretion signal peptide of OsmY (osmotically-inducible protein Y) was used to replace the *araBAD* genes on the chromosome of TA-1, creating the vaccine parental strain TA-2. After induction with L-arabinose and purification via density gradient centrifugation, the *H. pylori* vaccine TA-2m was harvested. To enhance the immune response elicited by TA-2m, a dendritic cell (DC)-targeting aptamer was coated onto the surface of TA-2m, generating the DC-targeted vaccine Apt-TA-2m. These two vaccine candidates were then assessed for their safety, immunogenicity, and protective efficacy.

## Methods

### Cell culture and animals

The mouse dendritic cell (DC) line DC2.4 was purchased from the Cells Bank of the Chinese Academy of Sciences. Female BALB/c mice, 6-8 weeks old, were purchased from Chengdu Dashuo Laboratory Animal Co., Ltd.

Cells of DC2.4 were cultured in RPMI 1640 medium containing 10% fetal bovine serum (FBS) and 1% penicillin/streptomycin (Gibco, MA, USA). Mice were housed in a SPF-level animal room. All animal experiments were approved by the Laboratory Animal Committee of West China School of Public Health, Sichuan University (NO. Gwll2023212). The study adhered to the principles of the 3Rs: animals were only used when no suitable *in vitro* alternatives were available (Replacement), the minimum number of mice needed to achieve statistical power was used (Reduction), and all procedures were designed to minimize pain and distress (Refinement).

### Bacteria and growth conditions

All bacterial strains used in this study are listed in **Table 1**. *S. typhimurium* strain TH4531 (PKD4 LT2), TH4702 (PKD46 LT2), and TH6701 (Δ*araBAD*::*tetRA* LT2) were kind gifts from Prof. Kelly T. Hughes at the University of Utah. The *H. pylori* reference strain SS1 was purchased from American Type Culture Collection (ATCC). *S. typhimurium* strains were grown in Luria-Bertani (LB) broth or on LB agar. Antibiotics were added at the following final concentrations when needed: ampicillin at 100 μg/mL, tetracycline at 15 μg/mL, and kanamycin at 50 μg/mL. *H. pylori* Strain SS1 was cultured on blood agar plate supplemented with 7% defibrinated goat blood.

**Table 1:** Bacterial strains used in this study.

Strain	Genotype	Source
*S. typhimurium*		
TH4531	PKD4 LT2	Hughes KT
TH4702	PKD46 LT2	Hughes KT
TH6701	Δ*araBAD*::*tetRA* LT2	Hughes KT
TT-121	Δ*araBAD*::*tetRA* PKD46 LT2	This study
TT-122	Δ*araBAD*::*tetRA* Δ*minD*::*FKF* PKD46 LT2	This study
TA-1	Δ*araBAD*::*tetRA* Δ*minD*::*FKF* LT2	This study
TA-2	Δ*araBAD*::*osmY-C1* Δ*minD*::*FKF* LT2	This study
*H. pylori*		
SS1	wild-type	ATCC

### Construction of vaccine antigen

The MHC class II-restricted T-cell epitopes, B-cell epitopes, and peptide fragments enriched with both types of epitopes used in this study were derived from major antigenic proteins of *Helicobacter pylori*, including HpaA, VacA, and UreB. Most of these epitopes have been characterized previously (41), and are documented in the Immune Epitope Database (IEDB) with corresponding reference identifiers (RI). The peptide fragments were reported by Guo and colleagues (42). Additional MHC-II-restricted T-cell epitopes were predicted using the NetMHCIIpan 4.1 EL method available through the IEDB analysis resource, with the following parameters: (1) species/locus set to mouse, H-2-I; (2) MHC alleles selected as H2-IAd and H2-IEd; (3) peptide length set between 12 and 18 amino acids; and (4) peptides ranked by percentile score. Epitope conservation was evaluated using the IEDB Epitope Conservancy Analysis Tool (http://tools.iedb.org/conservancy/), and potential cross-reactivity with host proteins was assessed via BLASTp searches against the *Mus musculus* proteome. An epitope with 100% conservation across *H. pylori* strains, a percentile rank below 10% in MHC-II binding prediction, and no significant homology to *Mus musculus* proteins (E-values ≥ 0.01 and sequence identities ≤ 35%) was selected.

To construct a chimeric antigen for vaccine development, the selected epitopes and peptide fragments were assembled using appropriate linkers. Specifically, KK linkers were used to connect MHC class II–restricted T-cell epitopes, while GGGS linkers were employed to link B-cell epitopes, to join individual peptide fragments, and to bridge peptide fragments with MHC class II–restricted T-cell epitopes. The antigenicity of the resulting epitope combinations was estimated using the online tool of VaxiJen 2.0 (43). The allergenicity of the epitope combinations was determined using the online software of AllerTOP 2.0 (44). The biochemical characteristics of the epitope combinations, including molecular weight (MW), theoretical isoelectric point (pI), instability index, and grand average of hydropathicity (Gravy), were evaluated using ProtParam (45). Additionally, the solubility propensity of the epitope combinations was assessed using the online server of Protein-Sol (46). Finally, the optimal epitope combination, designated C1, was fused to the secretion signal peptide derived from the OsmY protein of *S. typhimurium* strain LT2 to construct a chimeric antigen, OsmY-C1, for subsequent vaccine development. The gene encoding OsmY-C1 was synthesized and cloned into the pUC57 plasmid (pUC57-OsmY-C1) by Tsingke Co. Ltd (Beijing, China). The structure diagram of OsmY-C1 is shown in **Supplementary Figure S1**.

### Construction of minicell and vaccine parental strains

To construct the minicell and vaccine parental strains, the P22-mediated generalized transduction and/or λ Red-mediated targeted mutagenesis were employed. In brief, the P22 phage lysates of TH4702 (PKD46 LT2) were transduced into TH6701 (Δ*araBAD*::*tetRA* LT2), selecting for ampicillin resistance. The resulting strain, namely, TT-121 (Δ*araBAD*::*tetRA* PKD46 LT2), was used as a recipient strain for the generation of minicell parental strain. Specifically, the forward primer MinD-For combined with reverse primer MinD-Rev were used to amplify the kanamycin resistance gene cassette *FRT-Kan^R^-FRT* (*FKF*), using TH4531 (PKD4 LT2) as a template. The PCR fragment was then used to replace the *minD* gene in TT-121 via electroporation, selecting for kanamycin resistance. The newly formed *minD* mutant was the minicell parental strain TA-1 (Δ*araBAD*::*tetRA* Δ*minD*::*FKF* LT2) (**Table 1**).

To construct the vaccine parental strain, an intermediate strain, designated TT-122 (Δ*araBAD*::*tetRA* Δ*minD*::*FKF* PKD46 LT2), was generated by P22-mediated generalized transduction, using TH4702 (PKD46 LT2) as the donor strain. The primer pair of araD-C and araB-C was used to amplify the *osmY-C1* gene (the encoding gene of OsmY-C1) using plasmid pUC57-OsmY-C1 as a template. The PCR fragment was then used to replace the *tetRA* gene in TT-121 via electroporation, selecting for fusaric acid resistance (47). The resulting strain was the vaccine parental strain TA-2 (Δ*araBAD*::*osmY-C1* Δ*minD*::*FKF* LT2) (**Table 1**). The details of PCR primers are listed in **Supplementary Table S1**.

### Minicell purification and characterization

The minicells were purified using protocols described previously with minor modifications (48). Briefly, a single colony of TA-1 or TA-2 was grown overnight in 20 mL of LB broth at 37°C with agitation. Then, 10 mL culture of TA-1 was diluted in 1L of LB broth while TA-2 diluted in 1L of LB broth supplemented with 0.2% of L-arabinose (w/v). Both diluent cultures were grown at 37°C with agitation until the optical density at 600 nm (OD600) reached 1.2. The minicells were enriched in a series of two low-speed centrifugations at 2,000 g in which the supernatants were collected. Followed by passing through a 0.8 μm aqueous filter, the filtrate (crude extract) which containing minicells was further purified by a two-round of 5%-20% OptiPrep density gradient centrifugation, each at 2,000×g at 4°C for 20 min, and only the 5% OptiPrep top layer was collected. After washing twice with PBS, the purified minicells were subjected to transmission electron microscope (TEM) and dynamic light scattering (DLS) analysis.

For TEM, 5 μL of minicell sample was pipetted onto a 400-mesh copper grid. Followed by natural drying in a darkroom, 5 μL of 2% phosphotungstic acid (w/v) was dripped onto the grid to stain the sample for 3 min. After removing the excessive dye, the morphology and size of minicells were characterized using a transmission electron microscope (Tecnai G2 F20 S-TWIN, FEI, USA). For DLS analysis, 1 mL of minicell sample (0.1 mg/mL) was added to the cuvette or electrode cell. Follow by analyzing on a Zetasizer Nano ZS analyzer, the hydrodynamic diameter, particle dispersion index (PDI), and zeta potential of minicells were determined.

To estimate the number of parental cells in the purified minicell samples, both direct counting and standard plate counting methods were implemented. The protocol for the plate numeration method was described previously (49). In brief, serial tenfold dilutions of the minicell preparation were plated on LB agar plates and incubated overnight at 37 °C. The number of colony-forming units (CFUs) was then counted to determine the number of viable parental cells. For the direct counting method, a Leica optical microscope was used. Specifically, a 10 μL minicell sample was added to a Petroff-Hausser chamber, followed by counting the particles under 100×oil immersion objective. The total cell number was estimated according to the instructions of the manufacturer. The contamination rate (CR) of parental cells was calculated as follows: CR equals the number of viable cells divided by the total cell number. The number of viable cells was determined by the plate counting method, while the number of total cells were estimated by the microscope counting method.

### The preparation of DC-targeted *H. pylori* vaccine Apt-TA-2m

To enhance the immune response elicited by the minicell-based vaccine, DC targeted RNA aptamers (DC-Apt), which specifically recognize the DEC205 receptor on murine DCs (50), were synthesized and attached to the outer membrane of TA-2m. In brief, 100 μL of purified TA-2m (~2×10^5^) was incubated with 2.0 nM of DC-Apt at 4°C for 1 hour. Unbound aptamers were removed by ultrafiltration, and the minicells retained on the filter membrane were designated as Apt-TA-2m and subsequently resuspended in PBS (pH 7.0). To determine the minimal concentration of DC-Apt required for maximal loading (MML) on the minicell surface, a series of Cy3-labeled DC-Apt (Cy3-DC-Apt) dilutions, ranging from 0.05 nM to 2 nM, was prepared and tested using the same labeling procedure. The MML was identified as the lowest concentration of Cy3-DC-Apt that resulted in the minicells exhibiting maximum absorbance at 570 nm. The sequences of DC-Apt and Cy3-DC-Apt are listed in **Supplementary Table S2**.

### The uptake of Apt-TA-2m by DCs

Although the MML was determined, coating TA-2m with DC-Apt at the MML does not necessarily ensure maximum uptake by DCs. To address the minimal concentration of aptamers that leads to a saturated uptake by DCs, a serial concentration of DC-Apt around the MML ranging from 0 nM to 1.4nM were incubated with a fixed number (~2×10^5^) of fluorescein isothiocyanate (FITC) labelled TA-2m (FITC-TA-2m). For the uptake assay, the DC-Apt coated FITC-TA-2m were incubated with DC2.4 cells (1×10^5^) in a confocal dish at 37°C for 1 hour. Thereafter, cells were washed twice with PBS, and a staining solution containing 4’,6-diamidino-2-phenylindole (DAPI) and antifade mounting medium was added to each dish and incubated at room temperature for 5 min. The samples were then visualized using a A1 HD25 laser scanning confocal microscopy (Nikon, Kyoto, Japan).

### The location of antigen displayed by the minicell based vaccine

To investigate the location of the chimeric antigen in TA-2m, an osmotic shock method was employed to isolate various subcellular fractions (51). First, the crude extract of TA-2 or TA-1 was centrifuged at 16,800×g at 4°C for 20 minutes. The secreted proteins in the supernatant were collected. To isolate the periplasmic, cytoplasmic, and membrane proteins, cell pellets were resuspended in 7.5 mL of cell lysis buffer (20% sucrose, w/v; 30 mM Tris-Cl; 1 mM EDTA, pH 8.0) and incubated at room temperature for 10 minutes. Following centrifugation at 16,800×g at 4°C for 20 minutes, cells were resuspended in 7.5 mL of osmotic shock buffer (5 mM MgSO_4_) and incubated on ice for 10 minutes. The supernatant, containing the periplasmic proteins, was isolated by centrifugation and gently collected. The spheroplasts were resuspended in 1 mL of 0.1 M Tris-Cl (pH 8.0) and lysed through freeze-thaw cycles. After another round of centrifugation, the supernatant containing the cytoplasmic proteins was collected, while the membrane proteins in the precipitate were resuspended in 100 μL of 0.1 M Tris-Cl (pH 8.0). All subcellular extracts were subjected to protein precipitation and immunoblotting analysis.

For immunoblotting analysis, proteins were precipitated by the addition of 10% trichloroacetic acid (TCA) and centrifuged at 16,800×g at 4°C for 10 min. After washing twice with acetone and air-dried, the protein pellet was resuspended in a certain volume of PBS (pH 7.0) and subjected to a BCA kit (P0010S, Beyotime, Beijing, China) to determine the concentration. 10 μL of protein sample (1mg/ml) was run on a 12% tris–glycine polyacrylamide gel (PAGE) with 0.1% sodium dodecyl sulfate (SDS) added. Following SDS-PAGE, proteins were transferred to a polyvinylidene difluoride (PVDF) membrane. The membrane was blocked with Tris-buffered saline containing 0.1% Tween-20 (TBST) supplemented with 5% (w/v) non-fat dry milk for 1 h at room temperature. After blocking, the membrane was incubated overnight at 4 °C with rabbit anti-*H. pylori* antisera (1:50; LS-C58857, LSBio, Seattle, WA, USA). Following three washes with TBST, the membrane was incubated for 1 h at room temperature with horseradish peroxidase (HRP)-conjugated goat anti-rabbit IgG secondary antibody (1:1000; P0948, Beyotime, Beijing, China). Protein bands were visualized using NcmECL Ultra detection reagents (P2100, NCM Biotech, Suzhou, China) and imaged with the iBright FL1000 Imaging System (Thermo Fisher, MA, USA).

### Cell proliferation and toxicity test

100 μL of DC2.4 cells (1×10^5^ cells/mL) were seeded into a flat-bottom 96-well plate and allowed to adhere by incubation at 37°C for 24 hours with 5% CO_2_. Various numbers (2×10^4^, 2×10^5^, 2×10^6^, 2×10^7^, and 2×10^8^) of wild-type minicells, TA-2m or Apt-TA-2m were added to the wells, with wells containing only medium were served as the blank controls. Cells were then incubated at 37°C for 24 hours with 5% CO_2_. CCK-8 solution (Beyotime Biotech, Beijing, China) was added to each well at 10 μL/well, and the plates were incubated at 37°C for 4 hours in the dark. The optical density was measured at 450 nm (OD450) using a Multiskan GO microplate reader (Thermo Fisher, MA, USA), and the cell viability rate (%) was calculated using the following formula. Cell viability rate (%) = [(As-Ab)]/[(Ac-Ab)]×100%. ‘As’ represents the absorbance of experimental wells; ‘Ac’ represents the absorbance of negative control wells; ‘Ab’ means the absorbance of the blank control wells.

### *In vivo* safety assessment

#### Experiment 1

To estimate the median lethal dose (LD_50_) of the wild-type minicells and the minicell based vaccines, 96 mice were arbitrarily divided into three groups: the wild-type minicell group (Group 1), the TA-2 minicell group (Group 2); and the Apt-TA-2m group (Group 3). Each group was further divided into four subgroups, with each subgroup containing 8 mice (n=8). Prior to experiments, mice were acclimated for one week and fasted for 12 h, followed by oral lavage with 50 μL of 0.2 M NaHCO_3_ to neutralize the stomach acid. On day 0, each subgroup in Groups 1 to 3 was orally given 100 μL of PBS containing either 2×10^5^, 2×10^6^, 2×10^7^, or 2×10^8^ of wild-type minicells or TA-2m, or Apt-TA-2m. The LD_50_ was calculated using the Reed-Muench method (52), which determines the dose at which 50% of the animals succumb based on the proportion of survivors in each group across dose levels. Survival of the mice was monitored for fourteen consecutive days.

#### Experiment 2

To assess the pathologic damage caused by the wild-type minicells and the minicell based vaccines, 48 mice were arbitrarily divided into four groups, each containing twelve mice (n=12). On day 0, mice in group 1, 2, and 3 were orally administrated with 100 μL of PBS containing 2×10^8^ of wild-type minicells, TA-2m, and Apt-TA-2m, respectively. Group 4 is a control group and all mice in this group were served with 100 μL of PBS via the same route. At 1, 3, 5, and 7 days post-infection (dpi), three mice from each group were anesthetized with 2-4% isoflurane via inhalation. Once deep anesthesia was confirmed by the absence of a toe-pinch reflex, the animals were euthanized by cervical dislocation to ensure they were unconscious and did not experience pain or distress. Blood and major organ samples (liver, spleen, and kidney) were collected. The serum levels of aspartate aminotransferase (AST), alanine aminotransferase (ALT), blood urea nitrogen (BUN), and creatinine (CREA) were determined using a blood biochemical analyzer (BS360S, Mindray, China). The weight of each organ was measured, and the organ index calculated using the formula: Organ index = organ weight (g)/body weight (g).

For histopathological analysis, organs were fixed in 4% paraformaldehyde for 24 hours, followed by standard processing and Hematoxylin and Eosin (H&E) staining (53). Microscopic evaluation was performed, and tissue lesions were scored semi-quantitatively on a standardized scale from 0 to 4 based on the severity of pathological changes. The scoring criteria were as follows: 0 = no lesion, indicating no observable pathological changes; 1 = minimal, representing very slight alterations with no substantial impact on tissue function; 2 = mild, indicating evident changes with minimal disruption of tissue architecture; 3 = moderate, reflecting obvious structural alterations that may affect tissue function; and 4 = severe, representing extensive lesions with significant destruction of tissue architecture and likely impairment of normal function.

### Immune response

To explore the immune response elicited by minicell based vaccines, 32 mice were randomly assigned to four groups (Group 1 to Group 4), each containing eight mice (n=8). On Day 0, mice in Group 1 to Group 4 were orally administered 100 μL of PBS, or PBS containing 2×10^8^ of wild-type minicells, TA-2m, or Apt-TA-2m, followed by booster doses on day 14 and day 28, respectively. One weeks after the final immunization, mice in all groups were anesthetized and sacrificed. The blood, spleen, and fecal samples were collected and subjected to the subsequent immunological analyses immediately.

The titer of antigen-specific serum IgG and antigen-specific fecal IgA was measured using indirect ELISA assay. For IgG measurements, serum samples were directly applied to the ELISA, whereas for IgA quantification, 1 g of fecal samples was homogenized with 200 μL of PBS containing 2 mM PMSF, 50 mM EDTA, and 0.1 mg/mL soybean trypsin inhibitor, followed by centrifugation at 16,800 × g at 4°C for 10 minutes; the resulting supernatants were used in the assay. For the ELISA assay, each well in a 96-well plate was coated overnight at 4 °C with a B cell peptide pool consisting of UreB_327–334_, UreB_349–363_, HpaA_132–141_, VacA_1–46_, and VacA_332–494_ (each peptide at a final concentration of 0.4 µg/mL) in carbonate–bicarbonate buffer (pH 9.6). The plates were then washed with PBS containing 0.05% Tween-20 (PBST) and subsequently blocked for 1 hour at 37°C using 5% bovine serum albumin (BSA) in PBST to minimize nonspecific binding. After blocking, two-fold dilutions of serum samples (starting at 1:40) for IgG or the prepared fecal supernatants for IgA (staring at 1:5) were added to the wells and incubated for 1 hour at 37°C. Following sample incubation, the plates were washed thoroughly with PBST, and then incubated for 1 hour at 37°C with the horseradish peroxidase (HRP)-conjugated secondary antibodies (rabbit anti-mouse IgG at 1:2000 dilution for serum samples or rabbit anti-mouse IgA at 1:500 dilution for fecal supernatant). After another round of washing to remove unbound antibodies, 100 μL of 3,3′,5,5′-Tetramethylbenzidine (TMB) substrate solution was added to each well and allowed to develop in the dark at room temperature for 15 minutes. The reaction was terminated by adding 50 μL of 2 M H_2_SO_4_, and the absorbance was measured at 450 nm using a Multiskan GO microplate reader (Thermo Fisher, MA, USA). Antibody titers were determined based on the serial dilution of the samples and expressed as the reciprocal of the highest dilution yielding an optical density above the predetermined cutoff value.

To prepare single−cell suspensions, mouse spleens were gently homogenized through a 200−mesh nylon mesh and erythrocytes were lysed with an ammonium−chloride buffer. After two washes with RPMI 1640 medium, cells were resuspended in RPMI 1640 supplemented with 10% fetal calf serum. For flow cytometry analysis (FCA), splenocytes were plated at 2.5×10^6^ cells per well in a 96−well plate and stimulated for 5 hours at 37°C with a T−cell peptide pool comprising UreB_237–251_, HpaA_88–100_, HpaA_154–171_, VacA_1–46_, and VacA_332–494_ (each peptide at a final concentration of 2 µg/mL) in the presence of brefeldin A (final concentration at 5 µg/mL). Thereafter, cells were strained with FITC labeled Rat Anti-Mouse CD3, PercP labeled Rat Anti-Mouse CD4, and Brilliant Violet 605 labeled Rat Anti-Mouse CD8a antibodies in RPMI 1640 containing 1% fetal calf serum for 30 min at 4°C. Followed by permeabilization using a Cytofix/Cytoperm kit, the intracellular IFN-γ, IL-6, IL-4, and IL-17a were detected by straining with APC labeled Rat Anti-Mouse IFN-γ, PE/Cy7 labeled Rat Anti-Mouse IL-6, Brilliant Violet 421 labeled Rat Anti-Mouse IL-4, and APC/Cy7 labeled Rat Anti-Mouse IL-17a antibodies, respectively. Cells were fixed in 2% (w/v) para-formaldehyde and analyzed on a BD FACSverse flow cytometer. Unless otherwise specified, the Cytofix/Cytoperm kit and all antibodies were obtained from BD PharMingen™. All antibodies used for surface (extracellular) and intracellular staining were monoclonal and applied at a 1:100 dilution.

### Prophylactic vaccination

To determine how rapidly the minicell-based vaccines can confer protective immunity, 32 mice were enrolled and grouped as before. On Day 0, mice in Group 1 to Group 4 were orally vaccinated with 100 μL of PBS, or PBS containing 2×10^8^ of TA-1m, TA-2m, or Apt-TA-2m, followed by booster doses on day 14 and day 28, respectively. Two weeks after the final immunization (Day 42), mice in all groups were oral challenged with 1×10^9^ CFU of wild-type *H. pylori* SS1 four times in a week. Mice were then sacrificed two weeks after the last challenge (Day 63), a time point at which stable *H. pylori* colonization is established in naïve mice (54). The stomachs were collected for viable counting, urease test, and pathological analysis.

For bacterial numeration and urease test, the contents were taken from the stomachs and weighted. Followed by homogenization with PBS in a ratio of 1:0.5 (w/v), the viable number of *H. pylori* was determined using the *H. Pylori*-specific medium (HB8646a, Hopebio, Qingdao, China) supplemented with 1.5% agar. Whereas, urease in stomach homogenates was quantified using a urease detection kit (ml095000, Mlbio, Shanghai, China). For pathological analysis, the stomachs without contents were thoroughly rinsed with PBS and fixed with 4% paraformaldehyde for 24 hours. The tissue sections were stained with H&E, and the pathological changes were observed and scored.

### Statistical analysis

The statistical analysis was performed using IBM SPSS software ver. 22.0 (IBM, NY, USA). The differences of bacterial counts, antibody titer, cytokine concentration, and urease level between groups were evaluated using One-Way ANOVA and Tukey’s multiple comparison. Significance was taken as an adjust *p*-value of <0.05. The statistical charts were created using GraphPad Prism software ver. 8.0 (GraphPad Software, CA, US).

## Results

### Assembly of chimeric antigen

**Figure 1:**
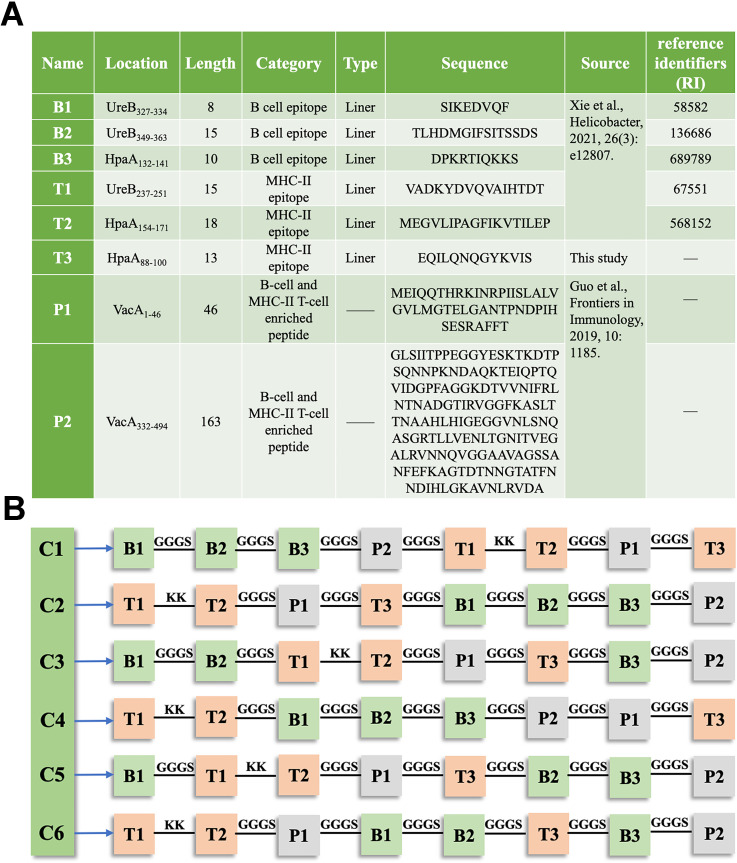
Composition of the chimeric antigen in TA-2m **(A)** Details of the epitopes and peptides used in the chimeric antigen, including their name, location, length, category, type, source, and amino acid sequences. **(B)** Schematic representation of the arrangement of epitopes and peptides within the chimeric antigen constructs.

In this study, a single MHC class II-restricted T-cell epitope, HpaA_88-100_, which met the prediction criteria, was identified using the IEDB analysis resource. The remaining epitopes and peptides were previously reported (**Figure 1A**). These epitopes and peptides were linked together using KK or GGGS linker to create six constructs. The arrangement of each epitope and peptide within these constructs is shown in **Figure 1B**.

The antigenicity, allergenicity, biochemical characteristics, and solubility of the constructs were evaluated using VaxiJen v2.0, AllerTOP2.0, ProtParam, and Protein-Sol, respectively. As shown in **Table 2**, all epitope combinations had antigenic scores (VaxiJen scores) above 0.8. The AllerTOP2.0 and ProtParam analysis indicated that these combinations were non-allergenic, each composed of 314 amino acids, with molecular weights ranging from 32.276 kDa to 32.506 kDa. The estimated pI values ranged from 8.91 to 9.38, with an instability index around 25. The gravy index varied from -0.384 to -0.427, and the solubility propensity index calculated by Protein-Sol was approximately 0.658 (**Table 2**). Among these, construct C1 presented similar allergenicity, solubility, and biochemical characteristics as the others but had a higher antigenic score (**Table 2**). Fusion of the OsmY secretion signal peptide to C1 (OsmY−C1) resulted in only minimal changes in physicochemical and antigenic properties (**Table 2**); therefore, OsmY-C1 was selected as the chimeric antigen for subsequent vaccine development. The amino acid sequences of OsmY signal peptide and C1 are detailed in **Supplementary Table S3**.

**Table 2:** The biochemical characteristics of the six epitope combinations.

Combination	VaxiJen score	Allergenicity	Amino acid composition	MV (kDa)	pI	Instability index	Gravy	Solubility propensity
C1	0.8796	Non-allergenic	314	32.506	8.91	25.43	-0.384	0.658
C2	0.8389			32.506	8.91	24.29	-0.384	0.658
C3	0.8628			32.388	8.91	24.02	-0.384	0.658
C4	0.8691			32.377	9.20	25.55	-0.384	0.659
C5	0.8289			32.276	9.20	24.02	-0.384	0.658
C6	0.8672			32.388	9.38	25.98	-0.427	0.658
OsmY-C1	0.8482		342	38.767	9.56	24.57	-0.390	0.623

### Characterization of minicells and minicell-based vaccines

**Figure 2:**
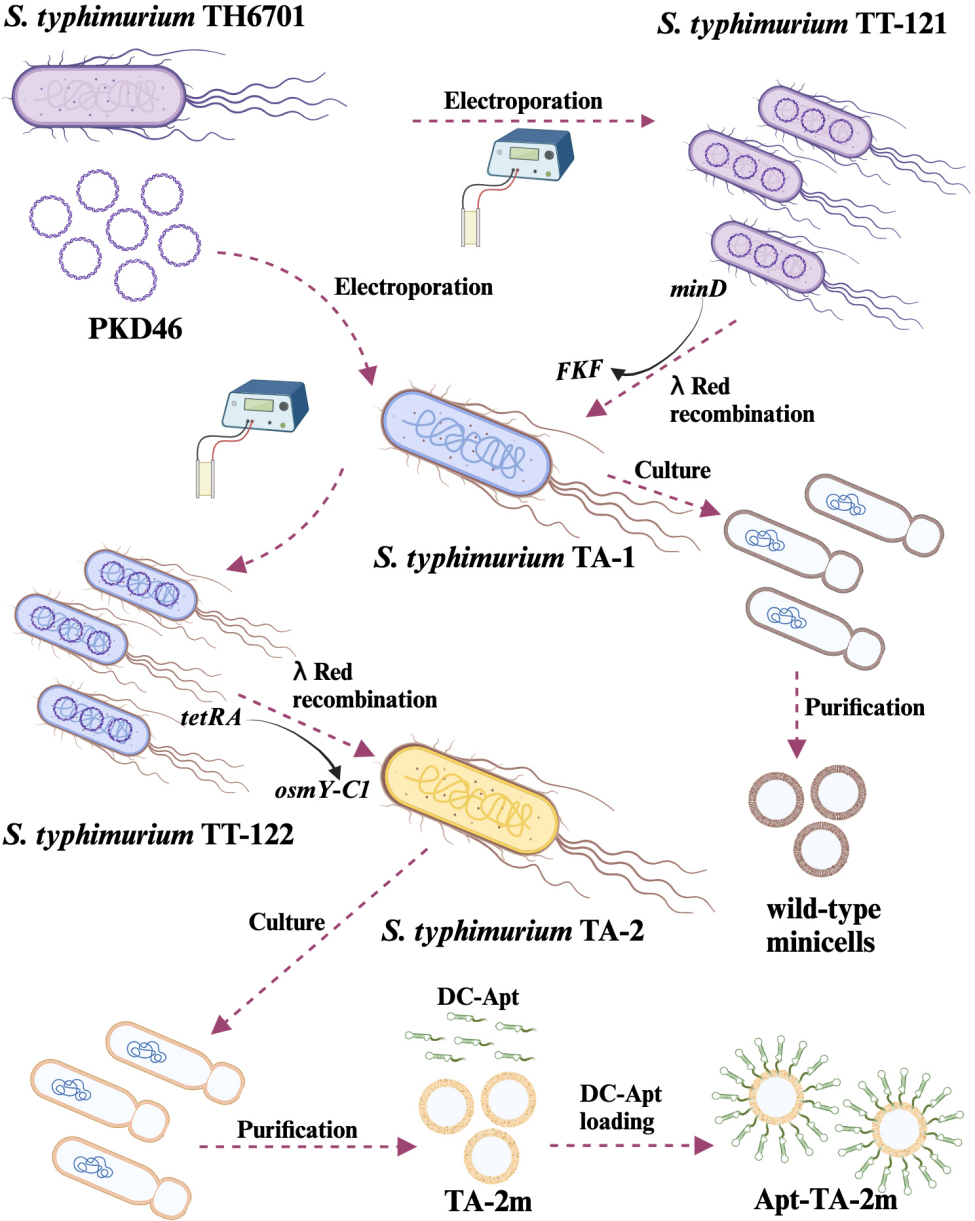
Schematic representation of genetically engineered bacterial minicell-based *H. pylori* vaccines. The minicell parental strain, TA-1, was created by replacing the *minD* gene with *FKF* in the TT-121. Wild-type minicells were isolated and purified from TA-1 cultures. The *H. pylori* vaccine parental strain, TA-2, was developed by replacing the *tetRA* gene of TA-1 with the gene encoding the chimeric antigen (OsmY-C1). Minicell-based *H. pylori* vaccine TA-2m was isolated and purified from TA-2 cultures, while the dendritic cell-targeted *H. pylori* vaccine, Apt-TA-2m, was constructed by coating TA-2m with the dendritic cell-targeting RNA aptamer (DC-Apt).

To elucidate the process of strain construction and vaccine preparation, a schematic diagram is presented in **Figure 2**. Initially, the wild-type minicell parental strain TA-1 (Δ*araBAD::tetRA* Δ*minD::FKF* LT2) was constructed. Subsequently, the OsmY-C1 encoding gene was introduced into TA-1 to replace the *tetRA* gene, resulting in the vaccine parental strain TA-2 (Δ*araBAD::osmY-C1* Δ*minD::FKF* LT2). Both TA-1 and TA-2 were cultured, and their derived minicells were isolated and characterized.

**Figure 3:**
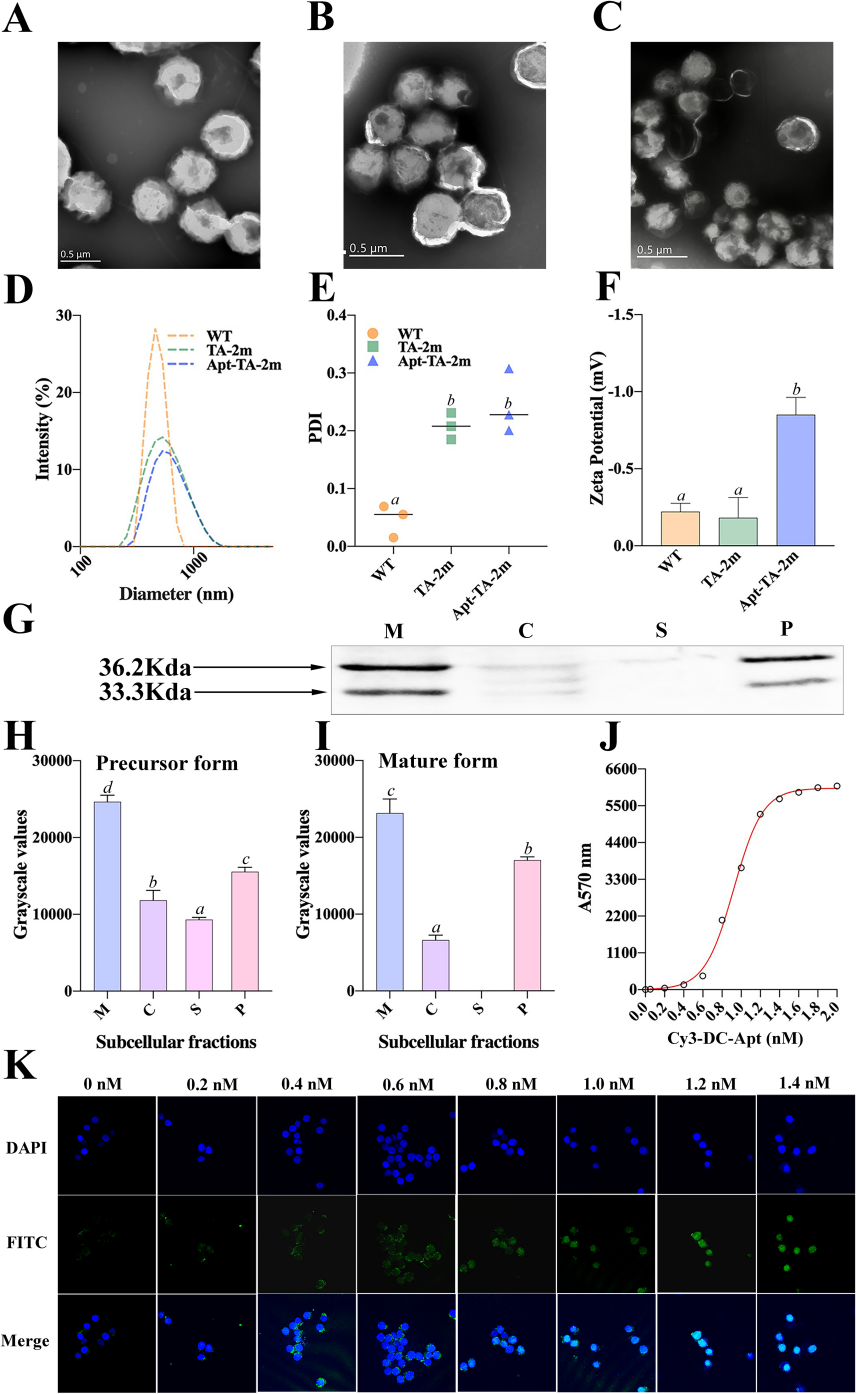
Characterization of wild-type minicells and minicell-based *H. pylori* vaccine constructs. **(A-C)** Transmission electron microscopy (TEM) images and schematic diagrams of wild-type minicells (WT), TA-2m, and Apt-TA-2m, respectively. **(D)** Size distribution of minicells determined by DLS. **(E)** Particle dispersity index of minicells determined by DLS. **(F)** Zeta potential of minicells assessed by DLS. **(G)** Immunoblot analysis of chimeric antigen expression in TA-2m. The capital letters M, C, S, and P denote the membrane, cytoplasmic, supernatant, and periplasmic fractions, respectively. **(H-I)** Relative expression levels of precursor and mature forms of the chimeric antigen in TA-2m, quantified by grayscale analysis of immunoblots. The capital letters M, C, S, and P denote the membrane, cytoplasmic, supernatant, and periplasmic fractions, respectively. **(J)** Loading curve of Cy3-labeled and cholesteryl-modified DC-Apt (Chol-Apt-Cy3) on TA-2m. **(K)** Uptake of TA-2m coated with varying concentrations of DC-Apt by DC2.4 cells. For all panels involving statistical analysis, significance was determined using One-Way ANOVA and Tukey’s multiple comparison with an adjusted *p*-value < 0.05. Groups labeled with the same letter (e.g., ‘a’ or ‘ab’) indicate no significant difference (*p* ≥ 0.05), while groups with different letters (e.g., ‘a’ vs. ‘b’) indicate significant differences (*p* < 0.05).

Either the wild-type minicells, TA-2m, or Apt-TA-2m exhibited spherical structure with a double-layered membrane (**Figures 3A–C**). The DLS results revealed that the mean diameter of wild-type minicells, TA-2m, and Apt-TA-2m were 459.6 nm, 531.2 nm, and 531.5 nm, respectively (**Figure 3D**). In addition to the size, DLS also determined the dispersion of minicells. The wild-type minicells had a PDI of 0.046, while the PDI of TA-2m and Apt-TA-2m were 0.208 and 0.246, respectively (**Figure 3E**). The successful loading of DC-Apt onto TA-2m was validated using DLS. Non-coated TA-2m exhibited a slight negative charge, with zeta potential value around -0.2 (**Figure 3F**). In contrast, minicells loaded with DC-Apt displayed a more negative charge, with zeta potential values around -0.8.

### Localization of the chimeric antigen in TA-2 minicells

A major goal of this study is to determine whether C1 fused with the OsmY signal peptide can be effectively delivered to the periplasm of minicells. To this end, minicells were extracted from strain TA-2, and the subcellular fractions of TA-2m were analyzed through SDS-PAGE and immunoblotting to determine the localization of the chimeric antigen.

To investigate the localization of the chimeric antigen in TA-2m, SDS-PAGE and immunoblotting analyses revealed two distinct bands at the expected molecular weights, representing different forms of the chimeric antigen: the precursor form (containing the signal peptide) at 36.2 kDa, and the mature form (lacking the signal peptide) at 33.3 kDa (**Figure 3G**). These bands were mainly found in the periplasmic and membrane fractions of TA-2m, indicating that the majority of chimeric antigen was successfully delivered into the periplasmic space or anchored on the membrane of TA-2m. The relative expression levels of the precursor and mature forms across different subcellular fractions are presented in **Figures 3H–I**.

### The loading of DC-Apt on TA-2 minicell and its uptake by DC2.4

To determine the minimal concentration of DC-Apt that results in maximum loading (MML) on minicells, a series of concentrations of Cy3-labeled DC-Apt (Cy3-DC-Apt) were incubated with a fixed number of TA-2m. The fluorescence intensity increased with the increasing concentration of Cy3-DC-Apt, reaching a plateau at 1.4 nmol (**Figure 3J**). Therefore, 1.4 nmol was considered the MML.

Although 1.4 nmol of aptamer led to maximal loading on minicells, it was unclear whether a higher loading of the aptamer on minicells would result in increased uptake by dendritic cells. As shown in **Figure 3K**, the number of minicells engulfed by DC2.4 cells increased with the increasing loading of aptamers, peaking at 1.2 nM, followed by a steady value. This phenomenon suggests that 1.2 nM is the minimal concentration of aptamers that leads to saturated uptake by dendritic cells.

### The safety of wild-type minicell and the minicell based vaccines

Since the parental strains of TA-1 and TA-2 are virulent, this study aimed to determine the contamination rate (CR) of the parental strain in purified minicell samples. Surprisingly, the CR for wild-type minicells was 0.030%, while for TA-2m, it was significantly lower at 0.003%, a 43-fold reduction. Besides the CR, cell proliferation and toxicity assays showed that neither wild-type minicells, TA-2m, nor Apt-TA-2m inhibited the growth of DC2.4 cells. Even at a high dosage of 2×10^8^, no reduction in cell viability was observed (**Supplementary Figure S2**).

**Figure 4:**
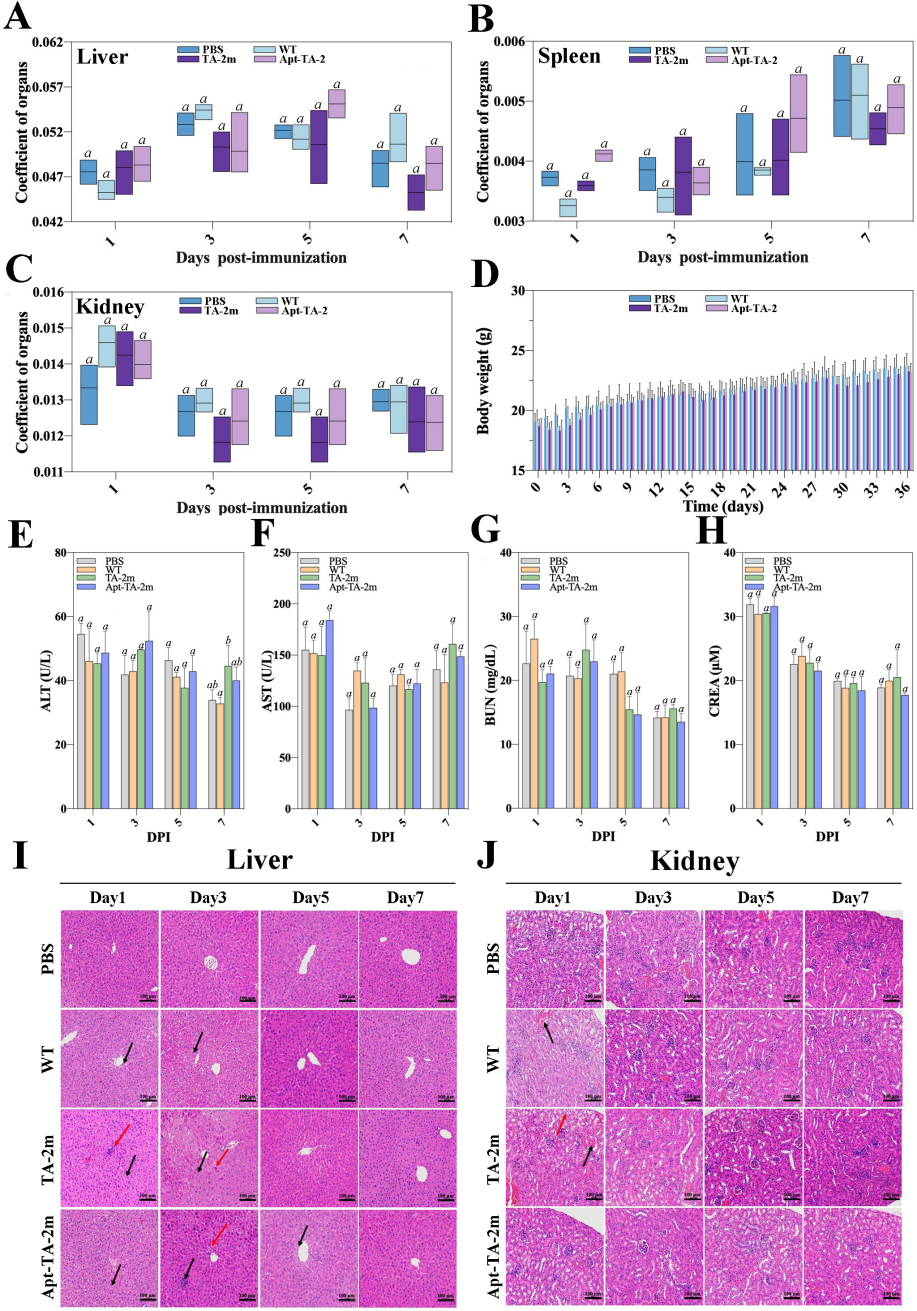
*In vivo* safety assessment of minicell-based *H. pylori* vaccines. Forty-eight female BALB/c mice (6–8 weeks old) were randomly assigned to four groups (n=12 per group). On Day 0, mice in Groups 1, 2, and 3 were orally administered 100 μL of PBS containing 2×10^8^ wild-type minicells, TA-2m, or Apt-TA-2m, respectively. Group 4 served as the control group and received 100 μL of PBS via the same route. At 1, 3, 5, and 7 days post-administration (dpi), three mice from each group (n=3) were anesthetized and assessed for liver **(A)**, spleen **(B)**, and kidney **(C)** organ coefficients, body weight **(D)**, and serum levels of ALT **(E)**, AST **(F)**, BUN **(G)**, and CREA **(H)**. Histopathological analyses of the liver **(I)** and kidney **(J)** were also conducted. In panel **(I)**, black arrows indicate hepatocellular hydropic degeneration, while red arrows highlight small focal infiltrates of inflammatory cells. In panel **(J)**, black arrows point to renal interstitial small veins, and red arrows indicate hydropic degeneration of renal tubular epithelial cells. For all panels involving statistical analysis, significance was determined using One-Way ANOVA and Tukey’s multiple comparison with an adjusted *p*-value < 0.05. Groups labeled with the same letter (e.g., ‘a’ vs. ‘a’, or ‘a’ vs. ‘ab’) are not significantly different (*p* ≥ 0.05), as they share at least one common letter. Conversely, groups labeled with different letters (e.g., ‘a’ vs. ‘b’) are considered significantly different (*p* < 0.05).

In the *in vivo* experiments, no mouse deaths were recorded in the LD50 assay, regardless of the dosage. The estimated LD50 value for wild-type minicells, TA-2m, or Apt-TA-2m was greater than 2×10^8^. To further investigate the toxicity of minicells, a fixed dosage (2×10^8^) of wild-type minicells, TA-2m, or Apt-TA-2m was orally administered to mice. Major organs (liver, spleen, and kidney) and blood were collected at 1, 3, 5, and 7 days post-inoculation (dpi). The organ coefficient data indicated no differences in the livers, spleens, and kidneys of immunized mice throughout the experimental period, regardless of the inoculum (**Figures 4A–C**). Similarly, almost no differences were observed in serum levels of ALT, AST, BUN, and CREA among the groups (**Figures 4E–H**).

However, the body weight of mice experienced a temporary decline after each immunization time point (0, 14, and 28 dpi) and returned to normal three or four days thereafter (**Figure 4D**). Additionally, the pathological examinations revealed minor impairments in the livers and kidneys of the minicell-treated groups (**Figures 4I–J**). Specifically, hepatocellular hydropic degeneration and small focal infiltration were observed in the livers at 1, 3, and 5 dpi. By day 7, liver impairments had returned to normal in all minicell-vaccinated groups (**Figure 4I**). Also, mice in the wild-type minicell group exhibited congestion of the renal interstitial small veins at 1 dpi. At the same time point, mice in the TA-2m group showed hydropic degeneration of the renal tubular epithelial cells and congestion of renal interstitial small veins. These conditions resolved by 3 dpi (**Figure 4J**). In contrast, the spleen of mice did not show any pathological changes, irrespective of groups and inoculum (**Supplementary Figure S3**).

### The immune responses elicited by the minicell based vaccines

**Figure 5:**
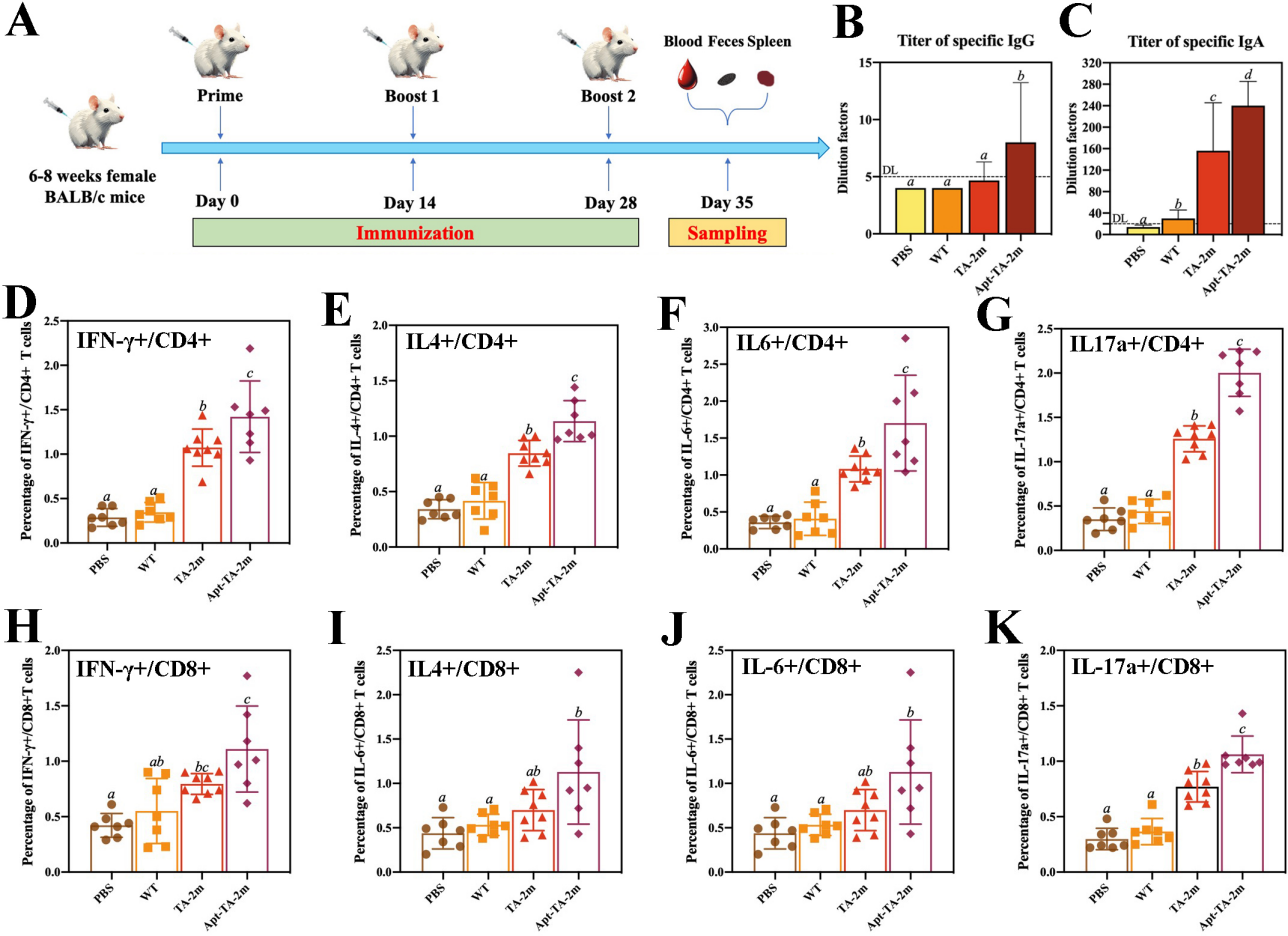
Humoral and T cell immunity induced by minicell-based *H. pylori* vaccines. Thirty-two mice were randomly divided into four groups (n=8 per group). On Day 0, mice in each group were orally administered 100 μL of PBS or PBS containing 2×10^8^ wild-type minicells, TA-2m, or Apt-TA-2m, followed by booster immunizations on Days 14 and 28. One week after the final immunization, all mice were anesthetized and sacrificed for immunological analyses. **(A)** Schematic representation of the immunization schedule and sampling time points. **(B, C)** Titers of serum specific IgG and fecal specific IgA antibodies, as measured by ELISA. **(D–G)** FCA-based quantification of CD4^+^ T cells producing IFN-γ, IL-4, IL-6, and IL-17A, respectively. **(H–K)** FCA-based quantification of CD8^+^ T cells secreting IFN-γ, IL-4, IL-6, and IL-17A, respectively. For all panels involving statistical analysis, significance was determined using One-Way ANOVA and Tukey’s multiple comparison with an adjusted *p*-value < 0.05. Groups labeled with the same letter (e.g., ‘a’ vs. ‘a’, or ‘a’ vs. ‘ab’) are not significantly different (*p* ≥ 0.05), as they share at least one common letter. Conversely, groups labeled with different letters (e.g., ‘a’ vs. ‘b’) are considered significantly different (*p* < 0.05).

A schematic of the immunization schedule and sampling time points is shown in **Figure 5A**. Serum and fecal specimens were collected at the indicated intervals, and an indirect ELISA was performed to quantify antigen−specific IgG and IgA titers. Serum IgG levels in the PBS, wild−type minicell, and TA−2m groups were comparable, whereas the Apt-TA-2m group induced a higher titer of specific IgG (**Figure 5B**), In contrast, IgA titers were higher in both the TA-2m and Apt-TA-2m immunized mice compared to those in the PBS and wild-type minicell groups (**Figure 5C**).

To assess the T cell immunity induced by the vaccines, we performed flow cytometry analysis (FCA) to quantify cytokine-producing T cells in vaccinated mice. Immunization with TA-2m and Apt-TA-2m significantly increased the frequency of CD4+ T cells producing IFN-γ, IL-4, IL-6, and IL-17a compared to mice treated with wild-type minicells or PBS (**Figures 5D–G**). Specifically, elevated IFN-γ production indicates a Th1-biased response critical for cellular immunity (55), while increased IL-4 suggests a Th2 response that supports humoral immunity (55). The enhanced IL-17a expression points to a Th17-mediated response, which may contribute to mucosal immunity and protection against extracellular pathogens (56). Notably, Apt-TA-2m further augmented the frequency of cytokine-producing CD4+ T cells, likely due to enhanced antigen presentation facilitated by DC-Apt loading. Similarly, CD8+ T cells from vaccinated mice exhibited significantly higher frequencies of IFN-γ- and IL-17a-producing cells compared to wild-type minicell or PBS group (**Figures 5H–K**), consistent with robust cytotoxic T lymphocyte (CTL) activity driven by IFN-γ, which is essential for eliminating infected cells. The detection of IL-17a-producing CD8+ T cells in Apt-TA-2m immunized mice may reflect a Tc17-like response that contributes to mucosal or tissue-specific immunity, as reported in models of viral and fungal infections (57, 58). The FCA scatter plots are provided in **Supplementary Figure S4**.

### Protective efficacy

**Figure 6:**
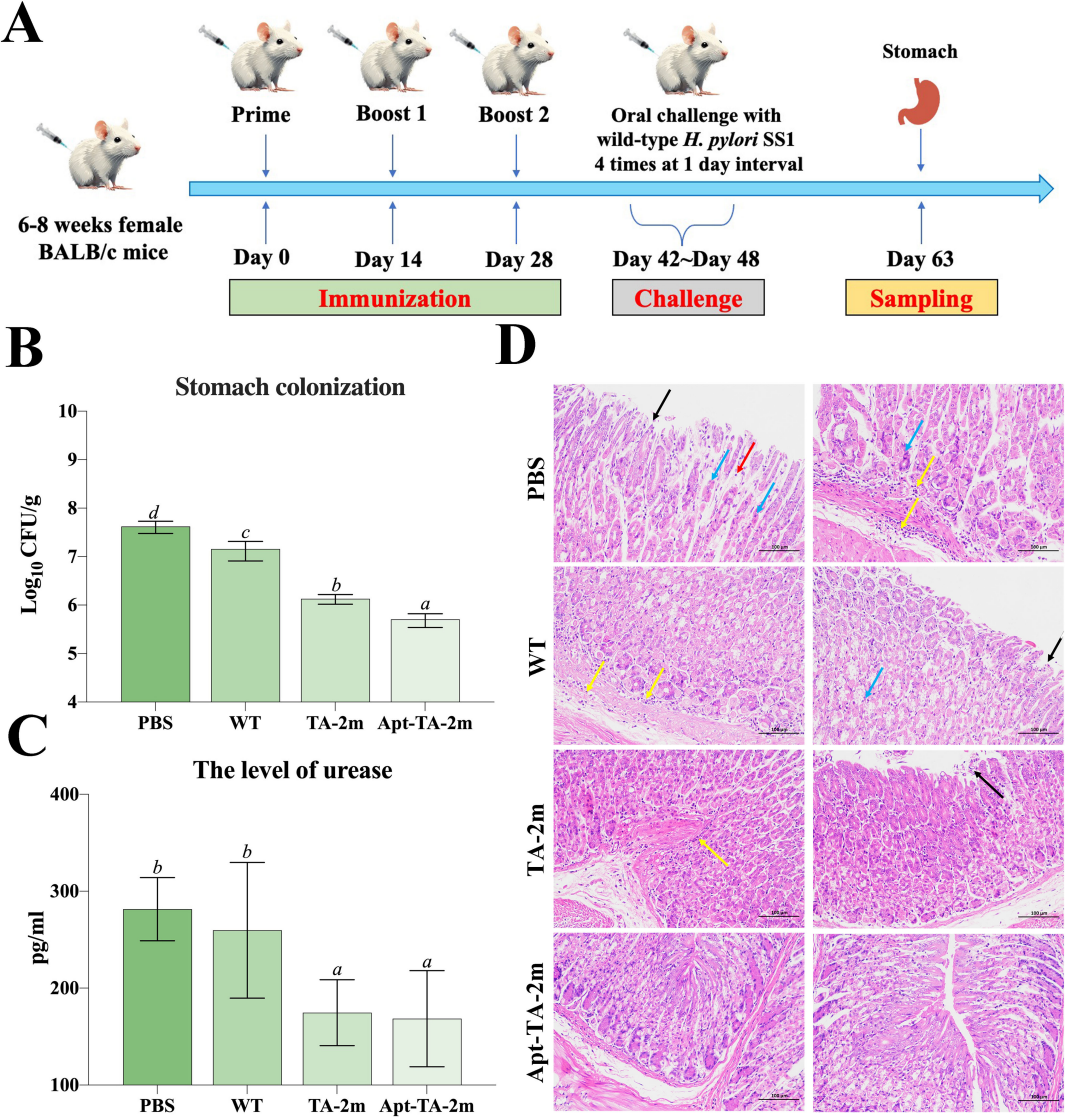
Immune protection induced by minicell-based *H. pylori* vaccines. Thirty-two female BALB/c mice (6–8 weeks old) were randomly assigned to four groups (n=8 per group). On Day 0, mice were orally immunized with 100 μL of PBS, or PBS containing 2×10^8^ of TA-1m, TA-2m, or Apt-TA-2m, followed by booster doses on Days 14 and 28. Two weeks after the final immunization (Day 42), all groups were orally challenged with 1×10^9^ CFU of wild-type *H. pylori* SS1, administered four times over one week. Mice were sacrificed two weeks after the final challenge (Day 63), at which point the stomachs were collected for bacterial load quantification by plate counting, urease activity assessment via ELISA, and histopathological evaluation. **(A)** Schematic overview of the immunization and challenge timeline. **(B)** *H. pylori* colonization levels in stomach tissues. **(C)** Urease concentration in the stomach. **(D)** Histopathological changes in the gastric mucosa. In panel **(D)**, black arrows indicate epithelial cell necrosis; blue arrows indicate gastric gland necrosis; red arrows show mild inflammatory cell infiltration, and yellow arrows denote areas with more extensive inflammatory infiltration. For all panels involving statistical analysis, significance was determined using One-Way ANOVA and Tukey’s multiple comparison with an adjusted *p*-value < 0.05. Groups labeled with the same letter (e.g., ‘a’ vs. ‘a’, or ‘a’ vs. ‘ab’) are not significantly different (*p* ≥ 0.05), as they share at least one common letter. Conversely, groups labeled with different letters (e.g., ‘a’ vs. ‘b’) are considered significantly different (*p* < 0.05).

The vaccination and challenge schedule is illustrated in **Figure 6A**. To assess the protective efficacy of the minicell-based vaccines, naïve mice and those immunized with wild-type minicells, TA-2m, or Apt-TA-2m were challenged with 1 × 10^9^ CFU of *H. pylori* SS1. Both TA-2m and Apt-TA-2m vaccination significantly reduced *H. pylori* colonization in the stomach, with the Apt-TA-2m group exhibiting the lowest bacterial load among all groups (**Figure 6B**). Consistently, gastric urease levels were significantly decreased in the TA-2m and Apt-TA-2m groups compared to the PBS and wild-type minicell groups (**Figure 6C**). Histopathological analysis further demonstrated that vaccination with TA-2m and Apt-TA-2m effectively protected the gastric mucosa from *H. pylori*-induced damage (**Figure 6D**). Mice in the PBS and wild-type minicell groups showed evident epithelial cell necrosis, partial gastric gland destruction, and substantial inflammatory cell infiltration. In contrast, TA-2m–immunized mice exhibited markedly reduced inflammatory infiltration and preserved epithelial architecture. Notably, no visible pathological alterations were observed in the gastric tissue of Apt-TA-2m–immunized mice following challenge (**Figure 6D**).

## Discussion

Epitope-based vaccines represent an innovative advancement over whole protein vaccines, retaining the most effective components to stimulate immune responses while minimizing the risk of non-specific reactions and side effects commonly associated with whole protein vaccines (27, 59). To construct an ideal epitope-based vaccine, it is crucial to identify and select appropriate T-cell and B-cell epitopes from major immunogenic proteins and develop effective strategies to deliver these epitopes.

This study designed the chimeric antigen to include MHC-II restricted T cell epitopes, B cell epitopes, and peptide fragments enriched with epitopes from *H. pylori* antigens (UreB, HpaA, and VacA) to induce a balanced immune response. These proteins were chosen as the sources of epitopes and peptides due to their protective roles and frequent use as candidate antigens in *H. pylori* vaccine development (60–68). HpaA, a surface adhesin, promotes bacterial colonization by binding gastric epithelial cells, making it a target for immune responses (67, 68). VacA induces vacuolation in host cells and modulates immune responses, with well-documented immunogenicity and the ability to induce neutralizing antibodies (65, 69). UreB, a subunit of urease, neutralizes gastric acid, enabling *H. pylori* survival. Its epitopes trigger robust humoral and cellular immune responses, offering protection in animal models (60, 62–64).

In addition to the selection of antigens, specific epitopes were chosen to optimize immune activation. The MHC-II restricted T cell epitopes were selected to induce CD4+ T cell-mediated Th1 and Th17 responses, which are critical for protective immunity against *H. pylori* by promoting bacterial clearance and reducing gastric inflammation (70–72). B cell epitopes were included to stimulate specific antibody responses, aiming to explore their potential role in enhancing immune synergy with T cell responses. However, recent studies suggest that *H. pylori*-specific antibodies may have limited effectiveness in bacterial eradication (73). To facilitate the immune processing of antigen, the KK linker was used to fuse the T cell epitopes, targeting cathepsin B, an essential protease involved in MHC-II restricted antigen presentation (74–76). Additionally, the GGGS linker was used to join the B-cell epitopes, individual peptide fragments, and bridge peptide fragments with T-cell epitopes. As a cleavable linker, GGGS separates the peptides from each other, promoting their expression (77, 78).

The molecular weight for all chimeric antigen constructs was estimated to be ~32 kDa, a desirable size since proteins with a molecular weight <110 kDa are considered suitable targets for vaccine production (79). The online server VaxiJen 2.0 was used to determine whether a protein sequence would be a protective antigen (43). All epitope combinations had a VaxiJen score above the default threshold of 0.4, indicating strong immunogenicity. Besides immunogenicity, other features such as allergenicity, biochemical characteristics, and solubility were also analyzed. The results from AllerTOP 2.0 demonstrated that all epitope constructs were non-allergenic, while the instability index calculated by ProtParam was around 25, suggesting that all constructs were stable since an index less than 40 indicates stability (80, 81). Regarding solubility, the estimated gravy index ranged from -0.384 to -0.427, indicating that all antigen constructs were hydrophilic (80). Among the constructs, C1 exhibited similar allergenicity, solubility, and biochemical characteristics as the others but had a higher antigenic score. Therefore, C1 was chosen as the optimal antigen construct.

Periplasmic display is an effective strategy to enhance the immune responses elicited by antigens (82). This strategy typically involves fusing the antigen to a secretion signal peptide, usually derived from a membrane protein like outer membrane protein A (OmpA) and alkaline phosphatase (PhoA) (83), or to an enzyme that targets the periplasmic space, such as β-lactamase (84). A previous study in *E. coli* demonstrated that the signal peptide of osmotically-inducible protein Y (OsmY) efficiently translocated fused heterologous proteins from the cytoplasm to the periplasm, achieving high yields (85, 86). This observation led us to hypothesize that such a peptide could perform a similar function in our vaccine strains, ultimately delivering the antigen to the periplasmic space of minicells. As expected, the fusion of the OsmY signal peptide effectively directed a substantial portion of the chimeric antigen to the periplasm in both its precursor and mature forms, rather than allowing it to accumulate in the cytoplasm, where it would typically localize in the absence of a secretion signal peptide.

Vaccine safety is a fundamental consideration in the process of regulatory approval and market authorization. Minicell-based vaccines, unlike live attenuated vaccines that retain limited replication capacity within the host, offer a significant safety advantage due to their lack of a chromosomal genome, thereby precluding replication (87). In the current study, no mortality was observed in mice administered the highest dose (2×10^8^) of wild-type minicells, TA-2m, or Apt-TA-2m, demonstrating the vaccines’ tolerability even at elevated concentrations. Although minor and transient histopathological changes were detected in the liver and kidneys of vaccinated mice, these alterations were not associated with functional impairments, as key biochemical markers of hepatic (ALT, AST) and renal (BUN, CREA) function remained within normal physiological ranges. While no significant differences in these markers were observed between treatment groups at each time point, some fluctuations occurred across sampling periods, which may be attributed to normal physiological variation, sampling inconsistencies, or minor deviations in assay performance. Importantly, the observed tissue changes resolved quickly, suggesting that any damage was superficial and reversible. Collectively, these findings support the favorable safety profile of the minicell-based *H. pylori* vaccines proposed in this study.

It has been suggested that the dendritic cells (DCs) play complex roles in *H. pylori* infection and immunity (88). On one hand, *H. pylori*-pulsed dendritic cells severely reduced the bacterial loads in the stomach of mice following a challenge with wild-type *H. pylori* (89, 90), indicating a critical role of DCs in priming protective immunity. On the other hand, DCs have been implicated in promoting tolerance to *H. pylori*, as a significant reduction in bacterial loads was observed in the stomachs of DC-depleted mice compared with wild-type mice (91). These multifaceted roles reflect the balancing act of DCs in managing *H. pylori* infection, prompting further investigation into whether a DC-targeted *H. pylori* vaccine could enhance or reduce immune protection. In this study, the DC-Apt-coated *H. pylori* vaccine Apt-TA-2m demonstrated a strong affinity for DC2.4 cells, as evidenced by a significantly higher uptake of Apt-TA-2m by DC2.4 cells compared to the ‘wild-type’ *H. pylori* vaccine TA-2m. This observation suggests that coating minicells with a DC-targeting aptamer may be an effective strategy to enhance dendritic cell (DC) targeting and uptake. Regarding the immune responses elicited by the minicell-based vaccines, both TA-2m and Apt-TA-2m were capable of inducing a Th1 cell-mediated immunity, as indicated by the increased numbers of CD4+ T cells producing IFN-γ. Notably, mice immunized with Apt-TA-2m exhibited a higher number of these T cells compared to those immunized with TA-2m. This suggests that incorporating a dendritic cell targeted aptamer (DC-Apt) into a minicell-based *H. pylori* vaccine could enhance its ability to induce a stronger T cell-mediated immune response. Expect for IFN-γ-producing Th1 cells, both vaccine candidates elicited a Th2 cell mediated immune response, as indicated by the increasing numbers of CD4+ T cells producing IL-4. Moreover, IL-17a, a pro-inflammatory cytokine, has been suggested to play a key role in enhancing the immune response against extracellular pathogens (92, 93). The increased numbers of CD4+ T cells producing IL-17a observed in TA-2m and Apt-TA-2m immunized mice suggests that both vaccine candidates are capable of triggering a Th17 response.

In addition to CD4+ T cell immunity, both vaccines elicited robust CD8+ T cell responses, evidenced by increased frequencies of CD8+ T cells producing IFN-γ+ and IL-17a. Elevated IFN-γ+ CD8+ T cells likely contribute to clearing *H. pylori*-infected gastric epithelial cells, a key mechanism in controlling chronic infection (94). The presence of IL-17A-producing CD8+ T cells (Tc17) in spleen and *H. pylori*-specific IgA in feces suggests potential induction of mucosal immunity (58). However, since *H. pylori*-specific IgA levels and Tc17 responses were not assessed in gastric tissue, the magnitude and nature of local mucosal immune responses remain unclear. Further studies focusing on both humoral and cellular immunity within the gastric mucosa are crucial to fully elucidate the protective mechanisms elicited by both vaccine candidates.

In agreement with immune responses triggered by the vaccine candidates, inoculation of TA-2m or Apt-TA-2m dramatically reduced the bacterial loads in the stomach. Specifically, the number of *H. pylori* SS1 in Apt-TA-2m immunized mice was lower than in TA-2m immunized mice, and 100-fold lower than in naïve mice, indicating that the loading of DC-Apt could further promote the immune efficacy of the minicell based *H. pylori* vaccine. It is important to note that the wild-type minicell vaccinated mice presented a lower bacterial load than the naïve mice. The causative reason is still unknown but one might suspect that the minicells per se could be an adjuvant to boost the immunity, since some popular vaccine adjuvants such as the Freund’s adjuvant harbored the bacterial components.

In conclusion, this study developed two multi-epitope vaccines against *H. pylori*: TA-2m and Apt-TA-2m, using *S. typhimurium*-derived minicells as a delivery platform. Both vaccine candidates demonstrated excellent safety profiles and elicited robust immune responses. Compared with previously reported *H. pylori* vaccines, this approach holds greater potential for clinical translation, largely due to the inherent safety advantages of minicells, which lack chromosomal DNA and are incapable of replication, thereby offering a safer alternative to live attenuated bacterial vectors. Moreover, the inclusion of a dendritic cell-targeting aptamer in Apt-TA-2m enhances antigen presentation and addresses the challenge of suboptimal immune protection. Taken together, these findings highlight the potential of DC-targeted, minicell-based vaccines as a promising and innovative strategy for the prevention of *H. pylori* infection.

## Data Availability

The original contributions presented in the study are included in the article/**Supplementary Material**. Further inquiries can be directed to the corresponding authors.
