# Supplementary material for: Construction and evaluation of a Salmonella minicell-based dendritic cell-targeted multi-epitope vaccine against Helicobacter pylori
Source: Front Immunol. 2025 Jun 5;16:1595096. doi: 10.3389/fimmu.2025.1595096 (PMC12176869; doi:10.3389/fimmu.2025.1595096)

## Supplementary Materials

**Table S1. The sequences of PCR primers used in this study**

| Name | Sequences (5' to 3') |
|---|---|
| MinD-For | ATCCCTTTTTAACAAGGAATTTTTATGGCACGCATTATT-GTGTAGGCTGGAGCTGCTTC |
| MinD-Rev | AAAATCCAGTAATGCCATAATTTATCCTCCGAACAGGCG-ATGGGAATTAGCCATGGTCC |
| araD-C | CCAGATTCATCAACGCGCCCCCCATGGGACGCGTTTTTAGAGGCA-TTAGTGGTGGTGGTGGTGG |
| araB-C | TCTCTACTGTTTCTCCATACCTGTTTTTCTGGATGGAGTAAGACG-ATGACTATGACAAGACTGAAGA |

**Table S2. The details of aptamer sequences and modification sites**

| Name | 5'- modify | 3'- modify | Sequences (5' to 3') |
|---|---|---|---|
| Cy3-DC-Apt | Cy3 | cholesteryl | Cy3-GGGAGGUGUGUUAGCACACGAUUCAUAAUCAGCUACCCUCCC-cholesteryl |
| DC-Apt | - | cholesteryl | GGGAGGUGUGUUAGCACACGAUUCAUAAUCAGCUACCCUCCC-cholesteryl |

**Table S3. The amino acid sequences of OsmY signal peptide and C1**

| Name | Amino acid sequence |
|---|---|
| OsmY signal peptide | MTMTRLKISKTLLAVMLTSAVATGSAFA |
| C1 | SIKEDVQFGGGSTLHDMGIFSITSSDSGGGSDPKRTIQKKSGGGSGLSIITPPEGGYESKTKDTPSQNNPKNDAQKTEIQPTQVIDGPFAGGKDTVVNIFRLNTNADGTIRVGGFKASLTTNAAHLHIGEGGVNLSNQASGRTLLVENLTGNITVEGALRVNNQVGGAAVAGSSANFEFKAGTDTNNGTATFNNDIHLGKAVNLRVDAGGGSVADKYDVQVAIHTDTKKMEGVLIPAGFIKVTILEPGGGSMEIQQTHRKINRPIISLALVGVLMGTELGANTPNDPIHSESRAFFTGGGSEQILQNQGYKVIS |

**Figure S1. The structure diagram of OsmY-C1. The red arrow indicates the OsmY signal peptide; the green arrow represents the peptide combination of C1; the purple arrow shows the location of His-tag; and the gray arrow indicates the termination codon.**

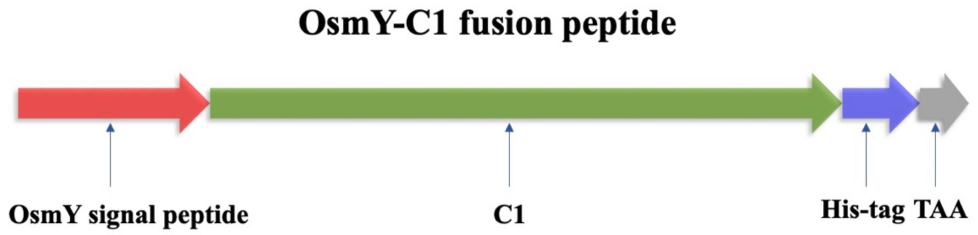


**Figure S2. Proliferation and cytotoxicity effects of wild-type (WT) minicells, TA-2m, and Apt-TA-2m on DC2.4 cells. DC2.4 cells were incubated with varying concentrations of WT minicells, TA-2m, or Apt-TA-2m at 37 °C for 24 hours with 5% of $CO_2$. Cell viability was assessed using the CCK-8 assay. Significance was determined using One-Way ANOVA and Tukey's multiple comparison with an adjusted *p*-value < 0.05. Groups labeled with the same letter (e.g., 'a' vs. 'a', or 'a' vs. 'ab') are not significantly different ($p \geq 0.05$), as they share at least one common letter. Conversely, groups labeled with different letters (e.g., 'a' vs. 'b') are considered significantly different ($p < 0.05$).**

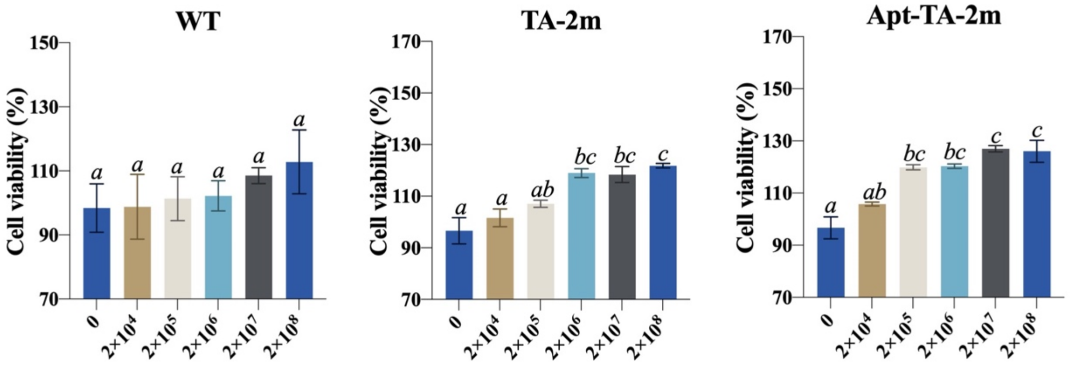

**Figure S3. Pathological changes in the spleens of mice immunized with the highest dose ($2\times10^8$) of wild-type minicells (WT), TA-2m, or Apt-TA-2m. A total of 48 female BALB/c mice (6–8 weeks old) were randomly divided into four groups (n = 12 per group). On Day 0, mice in Groups 1, 2, and 3 received an oral administration of 100 μL PBS containing $2\times10^8$ WT minicells, TA-2m, or Apt-TA-2m, respectively. Group 4 served as the control and received 100 μL of PBS via the same route. At 1, 3, 5, and 7 days post-immunization (dpi), three mice from each group (n = 3) were euthanized for histopathological examination. No significant pathological changes were observed in the spleens of any treatment group at the indicated time points.**

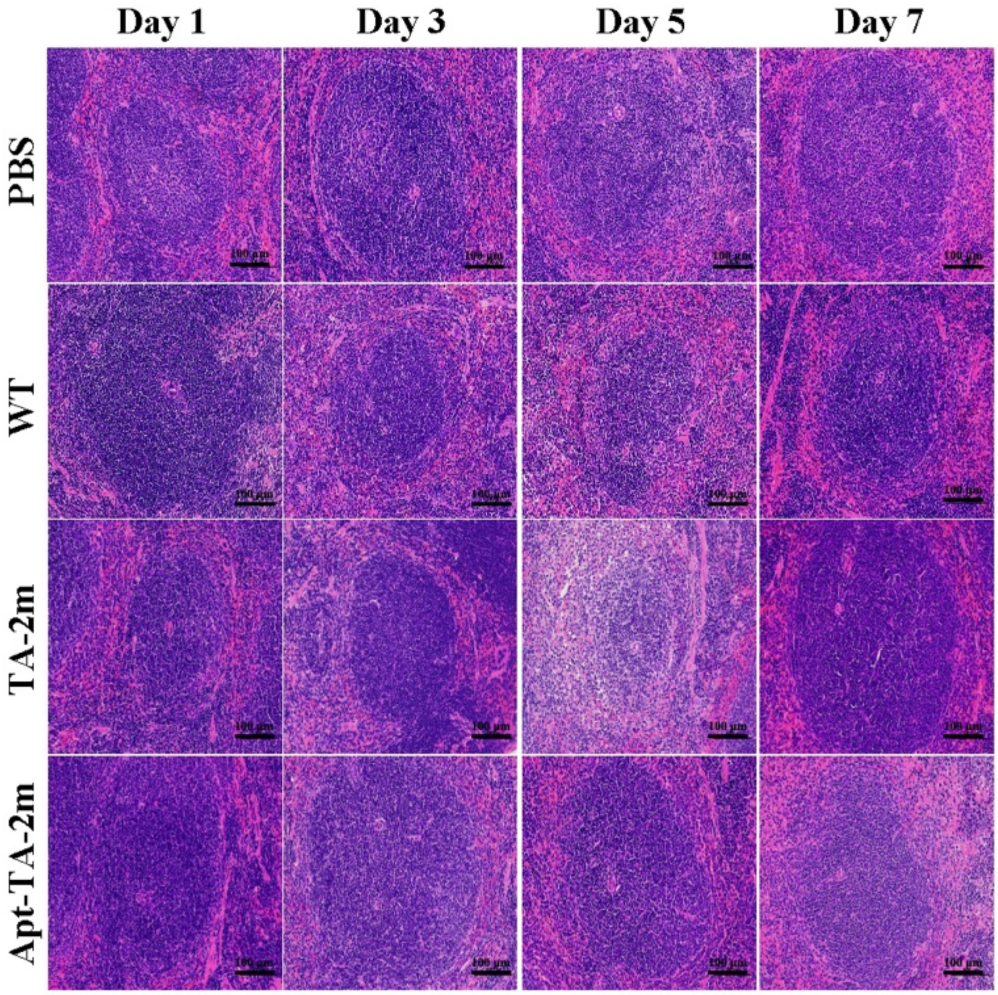

**Figure S4. The FAC scatter plots. Thirty-two mice were randomly divided into four groups (n=8 per group). On Day 0, mice in each group were orally administered 100 μL of PBS or PBS containing 2×10⁸ wild-type minicells, TA-2m, or Apt-TA-2m, followed by booster immunizations on Days 14 and 28. One week after the final immunization, all mice were anesthetized and sacrificed for FCA. (a) Percentages specific CD4+ T cells or CD8+ T cells producing IFN-γ. (b) Percentages specific CD4+ T cells or CD8+ T cells producing IL-4. (c) Percentages specific CD4+ T cells or CD8+ T cells producing IL-6. (d) Percentages specific CD4+ T cells or CD8+ T cells producing IL-17a.**

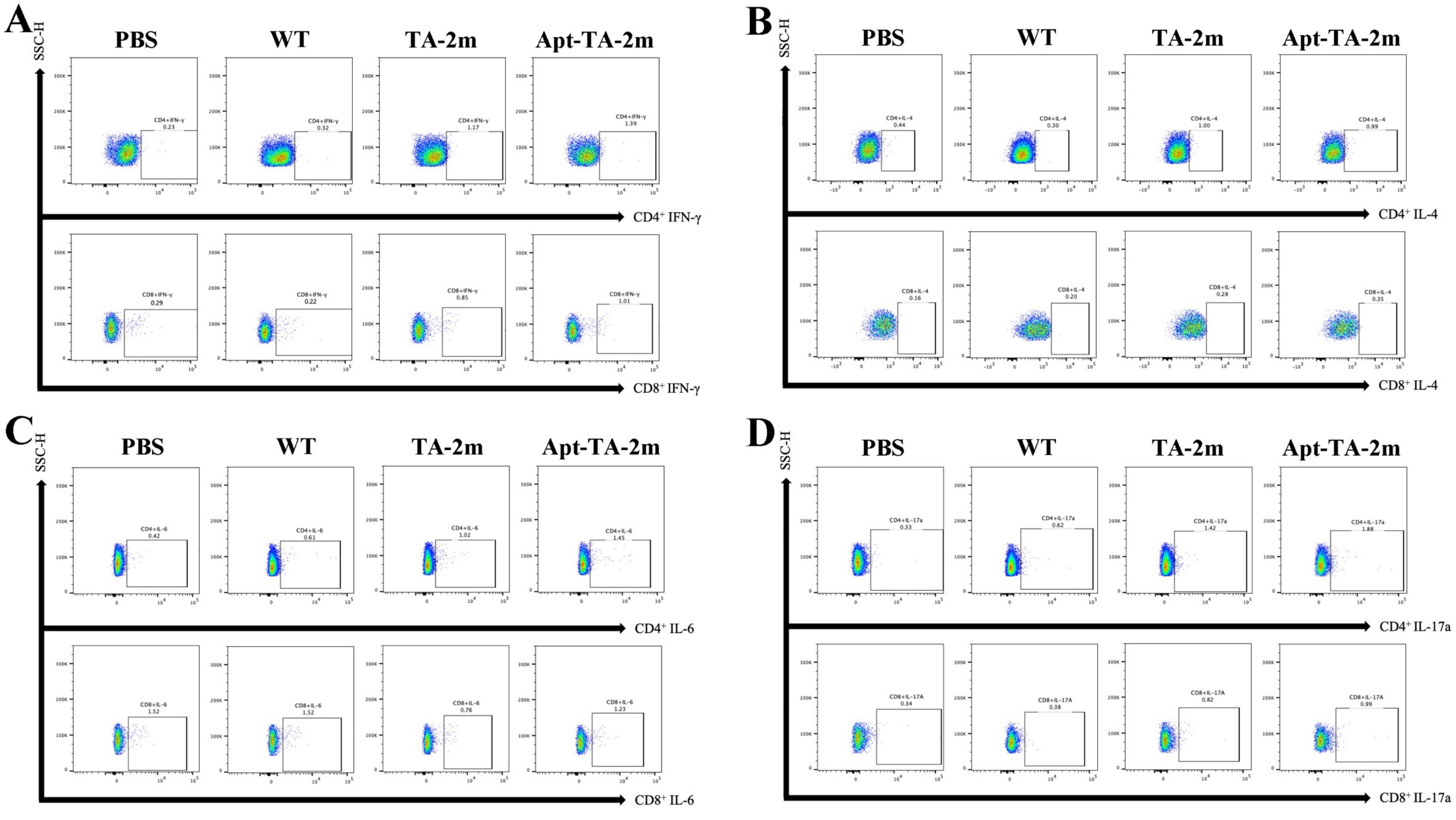

Supplement: Supplementary file 1 [file DataSheet1.pdf]
